# Prodrug–carboxypeptidase G2 therapy: certain concerns on carboxypeptidase G2

**DOI:** 10.3389/fphar.2025.1560834

**Published:** 2025-06-12

**Authors:** Tinghe Yu, Tianran Yu, Xinya Li

**Affiliations:** ^1^ Laboratory of Obstetrics and Gynecology, Second Affiliated Hospital, Chongqing Medical University, Chongqing, China; ^2^ Chongqing Yangjiaping High School, Chongqing, China

**Keywords:** prodrug-carboxypeptidase G2 therapy, pharmacokinetics, enzyme kinetics, antidrug antibody, therapeutic time window

## 1 Introduction

Prodrug–carboxypeptidase G2 (CPG2) is a promising anticancer strategy: clinical trials confirm that it can realize a targeted therapy, with a specific advantage of lacking human CPG2 analogues; further, preclinical trials demonstrate that it is effective against chemoresistant ovarian and lung cancers (Sharma and Bagshawe, 2017; Liu et al., 2020; Liu et al., 2022; Yu and Li, 2023; Qoura et al., 2024). CPG2 cleaves the amidic bond to release the active drug from the prodrug. Therefore, mutating or modifying CPG2 to improve the safety and efficacy is a hot topic, e.g., CPG2 is linked to an antibody or a ligand to realize the antibody/ligand directed prodrug therapy, and mutating CPG2 to alter the affinity or specific activity to a specific prodrug.

Native CPG2 (*Pseudomonas* sp. RS-16) has 23–415 and 26–415 variants due to removal of the signal peptide. These two forms have equal specific activities (400–600 U/mg) and K_m_ (8 μM), when using methotrexate (MTX) as the substrate. The catalytic domain contains 23–213 and 326–415 residues (Rowsell et al., 1997). Recombinant CPG2(26–415) has been clinically approved to rescue MTX intoxication; this form is therefore the preferred reference to determine the data reliability when developing next-generation CPG2.

## 2 Clinical info of licensed CPG2(26–415) (voraxaze)

Clinical data of voraxaze provide references for understanding the safety profile of CPG2. Voraxaze hydrolyzed MTX to decrease the toxic plasma MTX level in patients receiving high-dose MTX (>1 g/m^2^) such as treatments of osteosarcoma, leukemia and lymphoma, thereby rescuing MTX toxication (Bielack et al., 2024). The safety was verified in clinical trials and post-marketing experiences: no drug-related serious adverse events, and no common or very common adverse events were reported (US Center for Drug Evaluation and Research, 2012; EU Committee for Medicinal Products for Human Use, 2021).

Antidrug antibodies (ADA) were detected in 21% patients after injecting voraxaze. ADA did not impact on the safety profile, and was not a major concern because of the immunosuppressed status of cancer patients and a short treatment time (only one dose on most cases) (US Center for Drug Evaluation and Research, 2012). The findings are favorable to prodrug–CPG2 therapy, considering that immunogenicity of CPG2 is commonly considered as a defect (Sharma and Bagshawe, 2017; Yu and Li, 2023).

## 3 Concerns on mutated/modified CPG2 for prodrug–CPG2 therapy

Here analyses were based on mutated or modified CPG2 reported in 2000–2024. Those CPG2 molecules were developed for prodrug–CPG2 therapy, and therefore certain pharmacological and clinical concerns were discussed.

### 3.1 Affinity and catalytic efficacy

Specific activities of almost all novel CPG2 were not quantified, but which was a characteristic parameter of an enzyme (Supplementary Table S1). The CPG2 activity based on MTX utilizes the kinetic method, i.e., the reaction rate is linear in a short time that reflects the activity. However, the absorbance decrease within 10–60 min was used to compare activities between CPG2 in certain trials (Rashidi et al., 2018; AlQahtani et al., 2019; Al-Qahtani et al., 2019; Al-mansoori et al., 2020; Al-mansoori et al., 2021). The specific activity determines the CPG2 dose required for hydrolyzing an amount of prodrug, and can mirror the purity of CPG2 protein that impacts on the safety profile. Therefore, no specific activity is a major flaw.

CPG2 was mutated or modified to modulate the affinity. CPG2(23–415).A1extM-1;I99T, CPG2(23–415).A1extM-1;G122S or CPG2(23–415).A1extM-1;T328A had a higher affinity with K_m_ of 63–82 μM MTX, where K_m_ of CPG2(23–415).A1extM-1 was 172 μM (Al-Qahtani et al., 2019). K_m_ of pegylated Xen-CPG2 or human serum albumin–linker–Xen-CPG2 (70/66 μM MTX) was higher than that of Xen-CPG2 (51 μM; *Xenophilus azovorans* SN213), while K_m_ of CPG2(26–415).Q1extM-1 was 172 μM (Rashidi et al., 2018; AlQahtani et al., 2019). K_m_ demonstrated a lower affinity of CNGRC–Xen-CPG2–CNGRC, pegylated Xen-CPG2, pegylated CNGRC–Xen-CPG2 or pegylated CNGRC–Xen-CPG2–CNGRC (287–676 μM MTX) in comparison with Xen-CPG2 (236 μM) (Supplementary Table S1) (Al-mansoori et al., 2020; Al-mansoori et al., 2021). K_m_ of native CPG2 was >>8 μM MTX in those trials. Therefore, those data cannot be directly compared, and require particular concerns. Actual K_m_ of those CPG2 remain unclear. A drastic difference of K_m_ of Xen-CPG2 (4.7-fold) between trials indicates that novel CPG2 should be characterized under a standard operating procedure where the range of each character of the reference CPG2(26–415) should be preset.

K_cat_ is measured under full substrate saturation, reflecting the initial reaction rate (Davidia et al., 2016; Lu et al., 2017). Thus, K_cat_ cannot be used to predict the activation of prodrugs at therapeutic doses *in vitro* and *in vivo*. K_cat_ for MTX or ZD2767P was 10 or 30 s^-1^, respectively (Niculescu-Duvaz and Springer, 1996; Lee et al., 2023). In *in vitro* therapy, the catalytic efficacy of CPG2(26–415) for MTX or ZD2767P was 1 or 1/40 μmol/(min·U), respectively (Liu et al., 2020). Inconsistencies indicate that the catalytic efficacy to a specific prodrug should be specifically calibrated under therapeutic doses and conditions, with MTX as the reference. The time required for activating a definite amount of prodrug, and the yield of active drug after a definite time can be calculated:

$$\text{time required}=\text{amount of prodrug }/\;\left(\text{amount of CPG}2\times \text{catalytic efficacy}\right)$$

yield of active drug=amount of CPG2×catalytic efficacy×time

The amount of CPG2 required to activate a definite amount of prodrug in a definite time can be assessed:

$$\text{amount of CPG}2=\text{amount of prodrug }/\;\left(\text{catalytic efficacy}\times \text{time}\right)$$

Therefore, the catalytic efficacy of CPG2 to a prodrug is the critical parameter in formulating a therapy plan. Aforementioned verdicts can be used to predict the feature of intratumoral accumulation of active drugs vs. time (i.e., intratumoral pharmacokinetics (PK)), thereby assessing the pharmacodynamic effect, particularly when all independent variables in equations are characterized in cancer tissues (Zhang et al., 2017; Chang et al., 2023).

### 3.2 PK property

**FIGURE 1:**
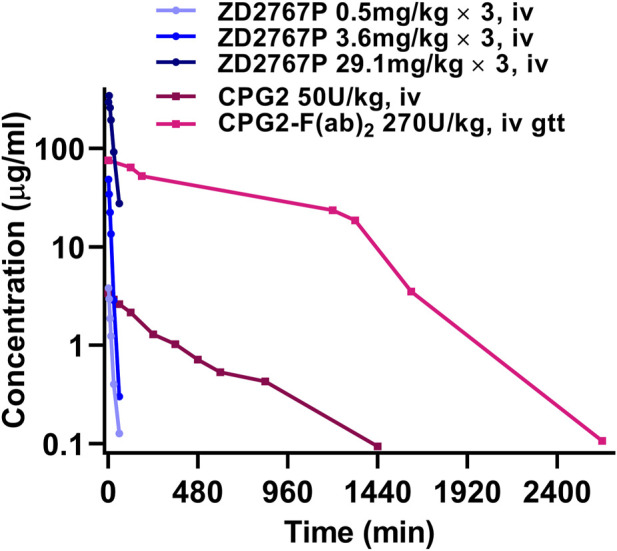
Blood drug level vs. time curves of ZD2767P and CPG2: PK property of CPG2 did not match with that of ZD2767P (data were from Napier et al., 2000; Francis et al., 2002; Mayer et al., 2006; Yu and Li, 2023).

The clinical PK property of CPG2 drastically differed from that of a prodrug (Figure 1). The half-life was 16 or 10 min for CMDA (4-[(2-chloroethyl) (2-mesyloxylethyl)amino]benzoyl-L-glutamic acid) or ZD2767P (4-[N,N-bis(2-iodoethyl)amino]phenoxycarbonyl-L-glutamic acid), and was 5 or 3 h for CPG2(26–415) or CPG2(23–415) linked to anti-carcinoembryonic antigen F(ab)_2_ antibody, respectively (Napier et al., 2000; Francis et al., 2002; Mayer et al., 2006; Yu and Li, 2023). Unmatched PK is a challenge: prodrugs should be administrated when the plasma CPG2 level decreases to a very low level to reduce off-target activation; a rapid elimination of prodrugs (although a short residence time favors the safety) indicates that enough amounts of prodrugs and CPG2 should be transferred into the tumor in a short time, i.e., a narrow therapeutic time window. The residence time of active drugs in cancer cells is longer that in plasma (e.g., ZD2767D (4-[N,N-bis(2-iodoethyl)amino]phenol), the active form of ZD2767P, had a half-life of ≈2 min in plasma, but the mean residence time was 14–28 min in cancer cells) (Yu et al., 2024). Therefore, more prodrugs should be activated and those active drugs should be released into cancer cells in a short time to combat the rapid elimination of prodrugs from blood and the tumor. These depend on the intratumoral total activity of CPG2 (amount × specific activity).

A narrow time window indicates that a higher yield rate of active drugs is favorable, depending on a higher specific activity of CPG2 (Liu et al., 2020). Unfortunately, specific activities of certain mutated/modified CPG2 were not quantitatively calibrated using CPG2(26–415) as the reference, limiting analyses of clinical potentials (Supplementary Table S1). Linking an antibody or a peptide to CPG2 decreases the specific activity, e.g., specific activities of CPG2, and CPG2 linked to anti-carcinoembryonic antigen single chain Fv antibody or to F(ab)_2_ antibody are 460, 128 and 80 U/mg, respectively (Yu and Li, 2023). Therefore, a higher dose of CPG2 conjugate is required to realize an equal total activity of CPG2.

Another PK-related issue is the ratio of CPG2 in tumor to blood, which determines the therapeutic precision. Intravenously infusing CPG2 linked to anti-carcinoembryonic antigen F(ab)_2_ antibody led to a median value of 0.4 (0–10.4) on prodrug day, i.e., low selectivity (Francis et al., 2002). The high variance is due to heterogeneity of *in vivo* distribution, and will complicate the outcome due to PK incoordination between CPG2 and prodrugs.

### 3.3 ADA against CPG2

ADA may impact on the behavior and activity of CPG2, and therefore reducing immunogenicity is in development (e.g., pegylation or modifying immunogenic epitopes) (Sharma and Bagshawe, 2017; Yu and Li, 2023; Qoura et al., 2024). A recent clinical trial demonstrated positive serum ADA at baseline in 3/20 cases, where the PK feature of CPG2(26–415) did not differ from that in cases with negative ADA. Further, CPG2(26–415) efficiently hydrolyzed MTX to reduce the plasma MTX concentration to a safe level in 1/4 ADA-positive patient. These data suggest that ADA against CPG2 may not be a critical concern for prodrug–CPG2 therapy. The catalytic domain of carboxypeptidase is highly conserved between species, and therefore human has partial tolerance to CPG2 (US Center for Drug Evaluation and Research, 2012).

### 3.4 His-tag and Met in the N-terminus

Most mutated/modified CPG2 was with poly(His) and/or Met in the N-terminus (Supplementary Table S1). The extension does not affect the specific activity, but may give rise to extra safety risks. Those CPG2 hardly have chances of being approved according to the drug regulations for therapeutic biologics (i.e., clinical futureless). Therefore, preclinical data using those CPG2 have poor clinical relevancies, and preclinical and translational trials should base on tag/Met-free CPG2.

## 4 Conclusion

CPG2 is the pivotal determinant in prodrug–CPG2 therapy. Intravenously injecting CPG2 or a CPG2 conjugate leads to a low amount of CPG2 in cancer tissues, although the expected goal of using a CPG2 conjugate is to realize an intratumoral enrichment of CPG2. Intratumoral application may be a solution considering the complexity of *in vivo* distribution. The catalytic efficacy to a specific prodrug should be specifically calibrated under therapeutic doses and conditions, and translational trials should utilize tag/Met-free CPG2.

## References

[B25] Al-mansoori L. Bashraheel S. S. Al Qahtani A. D. O'Connor C. D. Elsinga P. Goda S. K. (2020). In vitro studies on CNGRC-CPG2 fusion proteins for ligand directed enzyme prodrug therapy for targeted cancer therapy. Oncotarget 11, 619–633. 10.18632/oncotarget.27478 32110281 PMC7021235

[B1] Al-mansoori L. Al Qahtani A. D. Elsinga P. Goda S. K. (2021). Production of long-acting CNGRC-CPG2 fusion proteins: new derivatives to overcome drug immunogenicity of ligand-directed enzyme prodrug therapy for targeted cancer treatment. Technol. Cancer Res. Treat. 20, 15330338211057371. 10.1177/15330338211057371 34802309 PMC8606725

[B3] AlQahtani A. D. Al-mansoori L. Bashraheel S. S. Rashidi F. B. Al-Yafei A. Elsinga P. (2019). Production of “biobetter” glucarpidase variants to improve drug detoxification and antibody directed enzyme prodrug therapy for cancer treatment. Eur. J. Pharm. Sci. 127, 79–91. 10.1016/j.ejps.2018.10.014 30343151

[B4] Al-Qahtani A. D. Bashraheel S. S. Rashidi F. B. O'Connor C. D. Romero A. R. Domling A. (2019). Production of “biobetter” variants of glucarpidase with enhanced enzyme activity. Biomed. Pharmacother. 112, 108725. 10.1016/j.biopha.2019.108725 30970523

[B5] Bielack S. S. Soussain C. Fox C. P. Houillier C. Murciano T. Osborne W. (2024). A European consensus recommendation on the management of delayed methotrexate elimination: supportive measures, leucovorin rescue and glucarpidase treatment. J. Cancer Res. Clin. Oncol. 150, 441. 10.1007/s00432-024-05945-6 39356310 PMC11446969

[B6] Chang H. P. Le H. K. Shah D. K. (2023). Pharmacokinetics and pharmacodynamics of antibody-drug conjugates administered via subcutaneous and intratumoral routes. Pharmaceutics 15, 1132. 10.3390/pharmaceutics15041132 37111619 PMC10142912

[B7] Davidia D. Noorb E. Liebermeisterc W. Bar-Even A. Flamholz A. Tummler K. (2016). Global characterization of *in vivo* enzyme catalytic rates and their correspondence to *in vitro* Kcat measurements. Proc. Natl. Acad. Sci. U. S. A. 113, 3401–3406. 10.1073/pnas.1514240113 26951675 PMC4812741

[B8] EU Committee for Medicinal Products for Human Use (2021). Voraxaze: assessment report.

[B9] Francis R. J. Sharma S. K. Springer C. Green A. J. Hope-Stone L. D. Sena L. (2002). A phase I trial of antibody directed enzyme prodrug therapy (ADEPT) in patients with advanced colorectal carcinoma or other CEA producing tumours. Br. J. Cancer 87, 600–607. 10.1038/sj.bjc.6600517 12237768 PMC2364249

[B10] Lee J. P. Corless B. C. Gardner T. J. Scheinberg D. A. Tan D. S. (2023). Development of a *p*-hydroxybenzyl-alcohol-linked glutamate prodrug for activation by *Pseudomonas* carboxypeptidase G2. Org. Lett. 25, 6295–6299. 10.1021/acs.orglett.3c02130 37602700 PMC10543097

[B26] Liu Q. Zhong X. Zhang Y. Li X. Qian G. Yu T. (2020). Ultrasound enhances ZD2767P-carboxypeptidase G2 against chemoresistant ovarian cancer cells by altering the intracellular pharmacokinetics of ZD2767D. Mol. Pharm. 17, 1922–1932. 10.1021/acs.molpharmaceut.0c00008 32302486

[B11] Liu Q. Li X. Luo Y. Wang H. Zhang Y. Yu T. (2022). Ultrasonically enhanced ZD2767P-carboxypeptidase G2 deactivates cisplatin-resistant human lung cancer cells. Oxid. Med. Cell Longev. 2022, 9191233. 10.1155/2022/9191233 36388164 PMC9652066

[B13] Lu J. Dong Y. Ng E. C. Siehl D. L. (2017). Novel form of the Michaelis-Menten equation that enables accurate estimation of (k_cat_/K_M_)*K_I_ with just two rate measurements; utility in directed evolution. Protein Eng. Des. Sel. 30, 395–399. 10.1093/protein/gzx012 28338799

[B14] Mayer A. Francis R. J. Sharma S. K. Tolner B. Springer C. J. Martin J. (2006). A phase I study of single administration of antibody-directed enzyme prodrug therapy with the recombinant anti-carcinoembryonic antigen antibody-enzyme fusion protein MFECP1 and a bis-iodo phenol mustard prodrug. Clin. Cancer Res. 12, 6509–6516. 10.1158/1078-0432.CCR-06-0769 17085666

[B15] Napier M. P. Sharma S. K. Springer C. J. Bagshawe K. D. Green A. J. Martin J. (2000). Antibody-directed enzyme prodrug therapy: efficacy and mechanism of action in colorectal carcinoma. Clin. Cancer Res. 6, 765–772. 10741695

[B16] Niculescu-Duvaz I. Springer C. J. (1996). Development of prodrugs for ADEPT (antibody-directed enzyme prodrug therapy). Expert Opin. Investg. Drugs 3, 289–308.

[B17] Qoura L. A. Morozova E. Ramaa С. S. Pokrovsky V. S. (2024). Smart nanocarriers for enzyme-activated prodrug therapy. J. Drug Target 32, 1029–1051. 10.1080/1061186X.2024.2383688 39045650

[B18] Rashidi F. B. AlQhatani A. D. Bashraheel S. S. Shaabani S. Groves M. R. Dömling A. (2018). Isolation and molecular characterization of novel glucarpidases: enzymes to improve the antibody directed enzyme pro-drug therapy for cancer treatment. PLoS One 13, e0196254. 10.1371/journal.pone.0196254 29698433 PMC5919439

[B19] Rowsell S. Pauptit R. A. Tucker A. D. Melton R. G. Blow D. M. Brick P. (1997). Crystal structure of carboxypeptidase G2, a bacterial enzyme with applications in cancer therapy. Structure 5, 337–347. 10.1016/s0969-2126(97)00191-3 9083113

[B20] Sharma S. K. Bagshawe K. D. (2017). Antibody directed enzyme prodrug therapy (ADEPT): trials and tribulations. Adv. Drug Deliv. Rev. 118, 2–7. 10.1016/j.addr.2017.09.009 28916498

[B21] US Center for Drug Evaluation and Research (2012). Voraxaze: cross discipline team leader review.

[B22] Yu T. Li X. (2023). Development of ZD2767P-carboxypeptidase G2-ultrasound therapy against cisplatin-resistant cancer. Front. Oncol. 13, 1151613. 10.3389/fonc.2023.1151613 37274240 PMC10233003

[B23] Yu T. Li X. Yu T. Chen M. Sun Y. Ran R. (2024). Intracellular pharmacokinetics of activated drugs in a prodrug-enzyme-ultrasound system: evaluations on ZD2767P+CPG2+US. ACS Med. Chem. Lett. 15, 739–745. 10.1021/acsmedchemlett.4c00071 38746880 PMC11089658

[B24] Zhang Y. Li J. Yu T. (2017). Pharmacokinetic profiles of cancer sonochemotherapy. Expert Opin. Drug Deliv. 14, 745–753. 10.1080/17425247.2016.1232248 27589927

