# Supplementary material for: Prodrug–carboxypeptidase G2 therapy: certain concerns on carboxypeptidase G2
Source: Front Pharmacol. 2025 Jun 12;16:1560834. doi: 10.3389/fphar.2025.1560834 (PMC12198612; doi:10.3389/fphar.2025.1560834)
Supplement: Supplementary file 1 [file Table1.docx]

**Table S1.** List of K_m_, K_cat_ and specific activity of mutated/modified CPG2 (2000–2024)

| Molecule | poly(His) | K_m_  (μM) | K_cat_  (s^-1^) | Specific activity | Ref |
| --- | --- | --- | --- | --- | --- |
| CPG2(23–415).A1extM-1 (MALAQKR…AGK)  (Pseudomonas sp. RS-16) | + | 171.7 ± 65.66 | 24.83 ± 0.91 |  | Al-Qahtani et al. Biomed Pharmacother 2019, 112: 108725. |
| CPG2(23–415).A1extM-1;I99T | + | 62.68 ± 10.26 | 26.11 ± 0.36 | ↑ |  |
| CPG2(23–415).A1extM-1;G122S | + | 71.38 ± 13.85 | 27.21 ± 0.46 | ↑ |  |
| CPG2(23–415).A1extM-1;T328A | + | 82.41 ± 15.07 | 26.93 ± 0.44 | ↑ |  |
| CPG2(26–415).Q1extM-1 (MQKR…AGK) | + | 171.7 | 24.83 |  | Rashidi et al. Plos One 2018, 13: e0196254. |
| Xen-CPG2 (MQKR…AGK)  (Xenophilus azovorans SN213)  (94% identity to CPG2(26–415)) | + | 50.5 | 11.49 |  |  |
| Xen-CPG2 | + | 50.56 ± 10.71 | 11.49 ± 0.19 |  | AlQahtani et al. Eur J Pharm Sci 2019, 127: 79. |
| pegylated Xen-CPG2 | + | 69.97 ± 17.12 | 9.76 ± 0.21 |  |  |
| HSA–linker–Xen-CPG2 | + | 66.14 ± 21.85 | 8.4 ± 0.24 |  |  |
| Xen-CPG2 (MALAQKR…AGKG) | + | 235.6 ± 12.1 | 35.64 ± 1.4 |  | Al-mansoori et al. Oncotarget 2020, 11: 619.  Al-mansoori et al. Technol Cancer Res Treat 2021, 20: 15330338211057371. |
| CNGRC–Xen-CPG2 (MCNGRCGGALAQKR…AGKG) | + | 171.7 ± 16.5 | 29.8 ± 2.06 |  |  |
| CNGRC–Xen-CPG2–CNGRC  (MCNGRCGGALAQKR…AGKGGGCNGRC) | + | 676 ± 24.07 | 72.3 ± 1.7 |  |  |
| pegylated Xen-CPG2 | + | 450 ± 10.55 | 52.25 ± 3.2 |  |  |
| pegylated CNGRC–Xen-CPG2 | + | 287.2 ± 18.7 | 33.36 ± 1.24 |  |  |
| pegylated CNGRC–Xen-CPG2–CNGRC | + | 304.7 ± 14.05 | 37.33 ± 2.7 |  |  |
| CPG2(23–415) | + | 44.54 |  |  | Etemadi et al. J Biomol Struct Dyn 2023, 41: 11463. |
| CPG2(23–415).A214_G323del  trCPG2 | + | 95.56 |  | ↓ |  |
| CPG2–VE-17 | + | 142.6 |  | ↓ |  |
| trCPG2–VE-17 | + | 93.31 |  | ↓ |  |
| CPG2(23–415) | – | 7.2 ± 1.0 |  |  | Spooner et al. Cancer Gene Ther 2000, 7: 1348. |
| CPG2(23–415).N222Q;N264Q;N272Q  CPG2(Q)3 | – | 10.4 ± 4.0 |  | ↓ |  |
| CPG2(23–415).N222Q;N264S;N272Q  CPG2(QSQ) | – | 58 ± 10.6 |  | ↓ |  |
| CPG2(23–415).N222Q;N264A;N272Q  CPG2(QAQ) | – |  |  |  |  |
| CPG2(23–415).N222Q;N264T;N272Q  CPG2(QTQ) | – | 125 ± 24 |  | ↓ |  |
| c-erb-B2(1–22)–CPG2(Q)3–c-erb-B2(636–686) | – | 54 ± 6.8 |  | ↓ |  |
| c-erb-B2(1–22)–CPG2(QSQ)–c-erb-B2(636–686) | – | 51 ± 7.8 |  | ↓ |  |
| c-erb-B2(1–22)–CPG2(QAQ)–c-erb-B2(636–686) | – | 69 ± 6.0 |  | ↓ |  |
| c-erb-B2(1–22)–CPG2(QTQ)–c-erb-B2(636–686) | – | 45 ± 1.9 |  | ↓ |  |
| c-erb-B2(1–27)–GS–CPG2(Q)3–EFH_6_AS | – | 29.5 |  |  | Spooner et al. Br J Cancer 2003, 88: 1622. |
| VEGF(-26–115)–GS–CPG2(Q)3–EFH_6_AS | – | 37.5 |  | ↑ |  |
| CPG2(23–415) (ALAQKR…AGK) | + | 23.39 |  |  | Sadeghian & Hemmati. Mol Biotechnol 2021, 63: 1155. |
| AcCPG2 (ASN…KNK)  (Acinetobacter sp. 263903-1)  (44.3% identity to CPG2(23–415)) | + | 44.99 |  |  |  |

HSA: human serum albumin; VEGF: vascular endothelium growth factor; VE-17: anti-VEGF-A binder
